# Dietary Arecoline Supplementation Enhances Flesh Quality, Muscle Growth, and Protein Deposition in Grass Carp (*Ctenopharyngodon idella*): Insights Into TOR and FOXO Signaling Pathways

**DOI:** 10.1155/anu/1084008

**Published:** 2026-08-26

**Authors:** Liang-Qin Wu, Na Yao, Wei-Dan Jiang, Pei Wu, Yang Liu, Hong-Yun Zhang, Yao-Bin Ma, Chang-Ran Xu, Qi-Yuan Chen, He-Qun Shi, Xiao-Qiu Zhou, Lin Feng

**Affiliations:** ^1^ Fisheries College, Sichuan Agricultural University, Chengdu 611130, China, sicau.edu.cn; ^2^ Fish Nutrition and Safety Production University Key Laboratory of Sichuan Province, Sichuan Agricultural University, Chengdu 611130, China, sicau.edu.cn; ^3^ Key Laboratory of Animal Disease-Resistance Nutrition, Ministry of Education, Ministry of Agriculture and Rural Affairs, Key Laboratory of Sichuan Province, Chengdu 611130, China, agri.gov.cn; ^4^ Aquatic Health and Intelligent Aquaculture Key Laboratory of Sichuan Province, Chengdu 610093, China; ^5^ Guangzhou Cohoo Biotech Co. Ltd., Guangzhou 510663, China

**Keywords:** arecoline, flesh quality, grass carp, muscle growth, protein synthesis and degradation

## Abstract

Arecoline is an alkaloid isolated from the plant betel nut, which exerts a wide range of physiological effects by stimulating muscarinic receptors. To explore the effect of arecoline on the flesh quality of fish, grass carp were fed different levels of arecoline (0, 0.5, 1.0, 1.5, 2.0, and 2.5 mg/kg diet) for 63 days. Key findings demonstrated that arecoline significantly alleviated postmortem pH decline, reduced cooking losses, and decreased lactic acid content. It also enhanced the nutritional value of muscle by increasing the content of essential amino acids (EAAs) and polyunsaturated fatty acids (PUFAs). Moreover, it improved muscle flavor characteristics by increasing the content of amino acids associated with umami. Additionally, appropriate levels of arecoline increased muscle fiber diameter, the frequency of fibers with diameters greater than 50 μm, and the mRNA levels of myogenic regulatory factors (MRFs). These findings suggest that arecoline promotes muscle growth in grass carp. We further found that dietary arecoline may promote muscle protein deposition through protein synthesis and the ubiquitin‐proteasome pathway (UPP). Our findings provide novel evidence that arecoline improves fish flesh quality. These findings offer practical insights for aquaculture nutrition and highlight arecoline’s potential as a functional feed additive.

## 1. Introduction

With the rapid growth of global aquaculture production, consumers have increasingly high requirements for the quality of aquatic products. Flesh quality has become a key factor determining their market competitiveness and farming economic benefits rather than being solely related to nutrition [1]. Flesh quality is affected by a series of comprehensive indicators, such as nutrient composition (e.g., crude protein and crude fat), flavor (e.g., amino acids), and physicochemical properties (e.g., pH, water holding capacity [WHC], etc.) [2, 3]. Recent studies suggest that plant‐derived bioactive compounds, particularly alkaloids, may influence muscle quality by modulating metabolic pathways [4, 5]. Arecoline is a pyridine alkaloid and the primary bioactive component of the betel nut (*Areca catechu* L.). This compound exhibits partial agonistic activity at muscarinic acetylcholine receptors. Although no studies have been reported in aquatic animals, research on terrestrial animals has shown that arecoline has various physiological effects, including promoting digestion by inducing contraction of the lower esophageal sphincter in pigs through stimulation of muscarinic receptors [6], improving the oxidative stress status of chicks infected with coccidiosis, and exerting anti‐inflammatory regulatory effects by reducing the release of proinflammatory factors [7], among others [8]. However, no studies have been reported on the effect of arecoline on muscle quality in animals.

Muscle growth is a vital determinant of muscle quality and is generally driven by two key processes: myofiber hyperplasia and hypertrophy [9]. Fish muscle growth is regulated by cyclin proteins, myogenic regulatory factors (MRFs), and myosin heavy chain (MyHC) [10, 11]. Despite the significance of these factors in muscle growth, the effects of arecoline on muscle growth in animals remain unknown. Studies in rats showed that arecoline could increase serum insulin levels [12], and insulin supplementation decreased the *myod* and *myog* levels in chick myoblast cells [13]. Meanwhile, previous research has demonstrated that arecoline stimulates catecholamine secretion in chromaffin cells of rats [14], and catecholamines activate the *myhc* gene in cardiac cells [15]. These findings indicate that arecoline may be associated with muscle growth and require further research.

Muscle formation in fish is heavily influenced by protein synthesis. The insulin‐like growth factor 1 (IGF‐1)/phosphatidylinositol 3‐kinase (PI3K)/target of rapamycin (TOR)/ribosomal protein S6 kinase 1 (S6K1)/eukaryotic translation initiation factor 4E‐binding protein 1 (4EBP1) pathway is a crucial mediator of protein biosynthesis, influencing muscle development [16]. Nonetheless, no studies have explored the impact of arecoline on protein synthesis in animal muscles. Previous research has shown that arecoline effectively increased choline levels in the mouse brain [17], and dietary choline reduced the expression of muscle TOR and increased the expression of 4EBP2 in Jian carp (*Cyprinus carpio var. Jian*) [18]. Furthermore, low concentrations of arecoline have been found to activate the phosphatidylinositol 3‐kinase/protein kinase B (PI3K/AKT) pathway in HepG5 cells [19]. These findings suggest that arecoline may influence protein synthesis pathways, potentially affecting muscle growth. These results highlight the impact of arecoline on protein synthesis in animals, and its underlying molecular mechanisms warrant further elucidation.

In addition to protein synthesis, protein degradation is important in overall protein turnover, contributing significantly to muscle quality. The ubiquitin‐proteasome system, the primary regulator of protein degradation, critically depends on the activity of forkhead box O (FOXO) transcription factors. Despite the importance of protein degradation in muscle maintenance, little information is available regarding the effects of arecoline on the ubiquitin‐proteasome pathway (UPP) in animals. Despite the importance of protein degradation in muscle maintenance, no studies have yet reported the effects of arecoline on the UPP in animals. However, studies in rats have shown that arecoline can increase plasma eNOS protein expression [20]. Moreover, nNOS regulates FOXO3a via nitric oxide (NO) production, leading to the upregulation of muscle atrophy markers such as MuRF1 and MAFbx [21]. These findings indicate that arecoline may influence protein degradation, potentially through the ubiquitin‐proteasome system, highlighting the need for further research into its effects on muscle protein turnover and its role in maintaining muscle quality.

Grass carp (*Ctenopharyngodon idella*) is an important species in global freshwater aquaculture, characterized by exceptional growth performance and cost‐effective market value in food production systems. Therefore, this study investigated the effects of arecoline on the flavor quality, physicochemical parameters, muscle growth, and protein deposition in grass carp muscle and elucidated its potential mechanisms. Additionally, this study suggests that arecoline may modulate the IGF‐1/AKT/TOR pathway and the ubiquitin‐proteasome system in fish muscle, providing a theoretical basis for further elucidating the functional role of arecoline in protein metabolism. Furthermore, this study determined the optimal supplementation level of arecoline for grass carp, providing essential guidance for fish feed formulation design.

## 2. Materials and Methods

### 2.1. Experimental Design

Table 1 displays the base diet formulation in detail. Six diets with equal nitrogen and lipid content were formulated. Arecoline was added to the basal diet at 0.0, 0.5, 1.0, 1.5, 2.0, and 2.5 mg/kg diet. The uniformly mixed raw material powder was processed into pellets using an F‐75 Dual‐screw Extruder. The resulting pellets were dried in a dryer, and their moisture content was tested. Finally, the feed was packed in airtight containers and stored at –20°C to preserve its nutritional quality.

**Table 1:** Ingredients and nutrient composition of the basal diet (as‐fed basis, %).

Ingredients	Content	Nutrient level	Content
Fish meal^a^	5.00	Moisture	10.96
Cottonseed meal^a^	10.58	Dry matter	89.04
Rapeseed meal^a^	8.40	Crude protein	26.14
Soybean meal^a^	20.00	Crude lipid	5.16
Fish oil	2.45	Organic matter	79.09
Soybean oil	1.51	Crude fiber	4.23
Butylated hydroxyanisole (99%)	0.02	Gross energy, MJ (kg)	16.96
Ca (H_2_PO_4_)_2_	1.38	n‐3 PUFAs	1.02
Flour	45.58	n‐6 PUFAs	0.94
Mineral premix^b^	2.00	Available P^f^, %	0.40
Vitamin premix^c^	1.00	Nitrogen free extract^f^	43.56
Choline chloride premix^d^	1.00	—	—
Thr (98.5%)	0.08	—	—
Arecoline premix^e^	1.00	—	—
Total	100.00	—	—

^a^Fish meal: Sichuan Huayu Trading Co., Ltd.; Cottonseed meal: Sichuan Sanjiu Runyi Trading Co., Ltd.; Rapeseed meal: Chengdu Xinxing Grain and Oil Co., Ltd.; Soybean meal: Sichuan Hongdabo Feed Co., Ltd.

^b^Provided the following per kilogram of mineral premix (g): MnSO_4_∙H_2_O, 2.66; MgSO_4_∙H_2_O, 256.79; FeSO_4_∙H_2_O, 12.61; ZnSO_4_∙H_2_O, 8.87; CuSO_4_·5H_2_O, 0.95; Ca (IO_3_)_2_, 1.56; yeast selenium, 13.65; all ingredients were diluted with corn starch to 1 kg.

^c^Provided the following per kilogram of vitamin premix (g): retinyl acetate, 0.193; vitamin *D*_3_, 0.204; DL‐A tocopherol acetate, 23.20; vitamin K_3_, 0.38; thiamine nitrate, 0.1137; riboflavin, 0.731; vitamin *B*_6_, 0.452; calcium‐D‐pantothenate, 4.203; niacin, 3.44; mesoinositol, 28.5; vitamin *B*_12_, 0.94; D‐biotin, 1.05; folic acid, 0.168; vitamin C acetate, 9.77; all ingredients were diluted with corn starch to 1 kg.

^d^Provided choline chloride at 261.95 g/kg premix, and the rest was diluted with corn starch to 1 kg.

^e^Provided arecoline at 0, 0.5, 1.0, 1.5, 2.0, 2.5 mg/kg premix for the six treatment groups, and the rest was diluted with microcrystalline cellulose.

^f^Available P and nitrogen free extract contents were calculated according to NRC (2011).

### 2.2. Feeding Management

The experiments were conducted at the Sichuan Agricultural University, China. Grass carp without visible trauma were purchased from a fishery. Following a 4‐week acclimatization period (with the same feed, feeding frequency, and water conditions as in the formal experiment), 450 current‐year grass carp (mean weight: 607.87 ± 1.82 g) were randomly divided into six groups (25 fish per group; each treatment was replicated three times). The fish were housed in 18 net cages and fed at 8:00, 11:00, 15:00, and 19:00 daily until satiation. Approximately 30 min after each feeding, the uneaten feed pellets on the feeding tray were carefully collected. The collected pellets were oven‐dried at 60°C to constant weight, and the dry weight of the uneaten feed was recorded to calculate the actual feed intake. The daily feed allowance was adjusted based on the previous day’s feed consumption to minimize feed waste. Throughout the 63‐day experiment, the water was changed regularly to maintain optimal conditions: temperature at 26.75 ± 2.3°C, pH within 7.5–8.0, and dissolved oxygen consistently above 6.0 mg/L.

### 2.3. Biochemical Analysis

Fish were anesthetized using a solution of 50 mg/L benzocaine and then euthanized at the experimental endpoint. Thereafter, muscle tissues were immediately removed and placed in an ice‐cold environment to minimize tissue degradation and preserve integrity [22]. The pH values of the muscle samples were recorded at two time points: immediately after tissue collection (0 h) and 24 h posteuthanization [23]. Heating the muscle samples determined cooking loss. The weight loss was recorded to determine the cooking loss. Meat tenderness was determined through shear force measurement using the Materials Testing Instrument. Hydroxyproline (HYP) and lactate (LD) levels were measured using kits by alkaline hydrolysis and colorimetry, respectively. Amino acid analysis was performed with an amino acid analyzer. Fatty acids were quantified using a gas chromatographic analyzer. Specific reagents, kits, and instruments used for biochemical analysis are provided in Table S1.

### 2.4. Electronic Tongue Analysis

The taste characteristics were assessed using an electronic tongue (ASTREE LS16/48, Alpha MOS, Shanghai, China) equipped with salty (CTS), sour (AHS), bitter (SCS), sweet (ANS), fresh (NMS), and two composite taste universal sensors (PKS and CPS). Before conducting the assays, the electronic tongue was activated and calibrated to ensure accurate measurements. Following the activation and calibration, 2.00 g of muscle tissue from grass carp was weighed, minced, and homogenized in 25 mL of distilled water. The homogenized mixture was allowed to stand for 15 min. The mixture was then centrifuged for 10 min to separate the solid particles. The resulting supernatant was then filtered and diluted with distilled water to bring the total volume to 100 mL. The sample solution was carefully transferred to a special beaker designed for the electronic tongue, and the taste test was conducted. During the analysis, the sample was alternated with distilled water to ensure accurate readings and to maintain sensor cleanliness between measurements.

### 2.5. Histological Examination

The fixed muscles soaked in 4% PFA were removed for embedding, sectioned using a Leica HistoCore MULTICUT microtome, and placed on polylysine slides to prevent tissue detachment. The tissue sections were H&E‐stained for histological evaluation. To assess the impact on muscle fiber development, ~300 adjacent myofiber diameters were measured in triplicate for each treatment group using a semiautomatic contouring method in Image‐Pro Plus 6.0.0.260. The muscle fiber diameters were classified into three categories based on length: <20 µm, 20–50 µm, and >50 µm. The distribution and frequency of different fiber sizes were analyzed across treatments.

### 2.6. Quantitative Real‐Time PCR Analysis

The procedure was performed as previously described [24]. Total RNA extraction was performed carefully to ensure high‐quality RNA isolation. Reverse transcription was performed using the PrimeScript RT kit, facilitating the conversion of RNA to complementary DNA (cDNA). Quantitative real‐time PCR was then performed using 2× AccuProof HiFi HotStart SuperMix (Dye Plus) (EXONGEN, Chengdu, China) on a fluorescence‐based real‐time qPCR system. *β-actin* and *gapdh* were selected as reference genes based on their proven stability in grass carp muscle tissue in our preliminary experiments and previous studies. These two genes were used as internal controls to normalize target gene expression, and the geometric mean of their Ct values was calculated for each sample to minimize potential variation. Data were analyzed by the 2^−ΔΔCt^ method, ensuring that the primer efficiency was nearly 100%. The primer sequences are provided in Table S2.

### 2.7. Western Blot Analysis

Following the procedure outlined in our past research [25], protein extraction was performed with RIPA Lysis Buffer, and equivalent protein quantities were separated via SDS‐PAGE, followed by transfer onto PVDF membranes using a wet transfer system. To minimize nonspecific binding, the membranes were blocked with BSA for 1.5 h. Primary antibody incubation was then performed overnight at 4°C (antibody details provided in Table S3). After washing, the membranes were treated with secondary antibodies for 2 h under gentle agitation. The results were analyzed with ImageJ 1.53 K, allowing for semiquantitative comparison of protein expression levels across experimental groups.

### 2.8. Statistical Analysis

The calculation formula is presented in Table S4. Statistical analyses were conducted using one‐way ANOVA with Duncan’s multiple range test in SPSS 22.0. Results are expressed as mean ± SD, and 95% confidence intervals for the mean differences are provided for all significant between‐group comparisons. A significance threshold of *p*  < 0.05 was applied.

## 3. Results

### 3.1. Effects of Arecoline on Flesh Quality Parameters in Muscle

**Figure 1:**
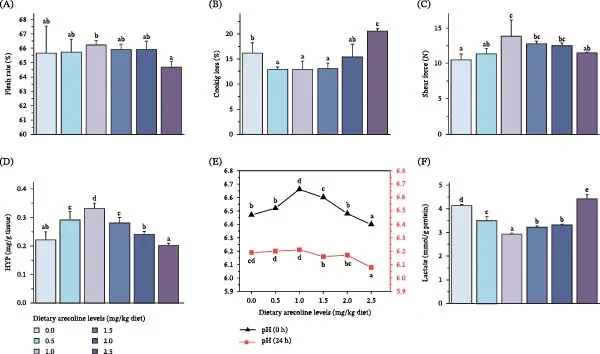
Effects of arecoline supplementation on the flesh quality parameters of the muscle tissues of adult grass carp. (A) Flesh rate (%); (B) cooking loss (%); (C) shear force (N); (D) HYP = hydroxyproline (mg/g tissue); (E) pH value of postmortem 0 and 24 h; and (F) lactate content (mmol/g protein). Values are means ± SD (*n* = 6). Different letters indicate significant differences (*p* < 0.05).

As shown in Figure 1, the 1.0 mg/kg group tended to have a higher flesh rate than the other groups. The 0.5 mg/kg arecoline group resulted in a significant reduction in cooking loss relative to the control (*p* < 0.001). Muscle shear force and HYP content showed significant increases at the 1.0 mg/kg arecoline group (*p* < 0.001). Two‐way ANOVA revealed significant main effects of diet and time, as well as a significant diet × time interaction on muscle pH (*p* < 0.001). Further pairwise comparisons at each time point showed that at 0 h postmortem, the 1.0 mg/kg arecoline group exhibited significantly higher pH values compared with the control group (*p* < 0.001). The lowest LD content in muscle was observed in the 1.0 mg/kg group (*p* < 0.001).

### 3.2. Effects of Arecoline on Amino Acid Composition in Muscle

**Figure 2:**
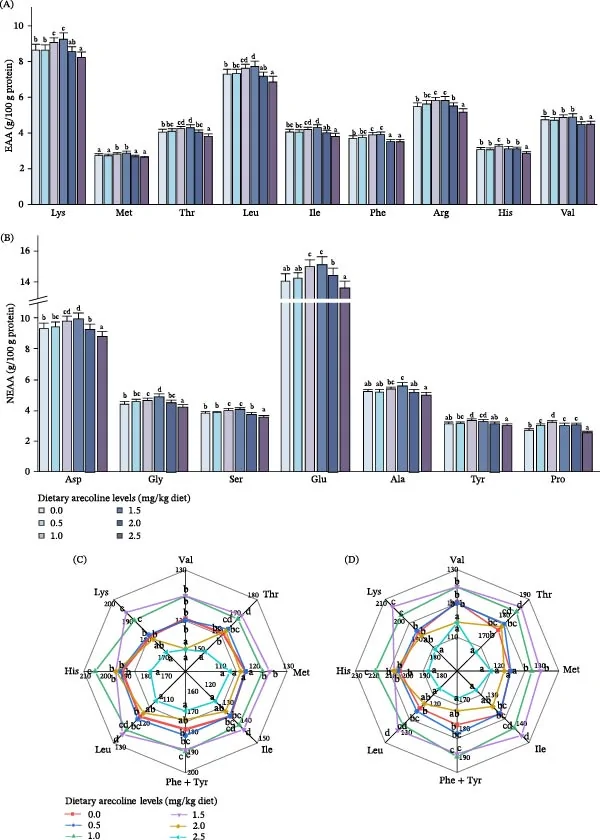
Effects of arecoline supplementation on the amino acid composition and amino acid chemical score in the muscle of grass carp. (A) The content of essential amino acids (EAAs); (B) the content of nonessential amino acids (NEAAs); (C) recommended EAA chemical scoring patterns for children 3–10 years old (% of scoring pattern); and (D) recommended EAA chemical scoring patterns for adults (>18 years old) (% of scoring pattern). The chemical score was calculated in relation to the reference protein pattern suggested by FAO/WHO/UNU. Values represent means ± SD (*n* = 6). Different letters indicate significant differences (*p* < 0.05).

The amino acid composition is shown in Figure 2A,B. The levels of proline (Pro), tyrosine (Tyr), and histidine (His) peaked in the 1.0 mg/kg arecoline supplementation group (*p* < 0.001). In addition, the concentrations of arginine (Arg), serine (Ser), glutamic acid (Glu), phenylalanine (Phe), methionine (Met), and lysine (Lys) were significantly increased in the 1.0 and 1.5 mg/kg arecoline supplementation groups (*p* < 0.001). The levels of amino acids such as threonine (Thr), aspartic acid (Asp), alanine (Ala), glycine (Gly), isoleucine (Ile), and leucine (Leu) were significantly elevated in the 1.5 mg/kg arecoline supplementation group (*p* < 0.001).

Figure 2C,D illustrate the effect of arecoline on the essential amino acid (EAA) score. Compared with the control, the chemical score of histidine (His) was significantly increased in the 1.0 mg/kg group (*p* < 0.001). Furthermore, 1.5 mg/kg arecoline significantly enhanced the chemical scores of threonine (Thr), leucine (Leu), and isoleucine (Ile) (*p* < 0.001), while methionine (Met), lysine (Lys), and phenylalanine plus tyrosine (Phe+Tyr) showed significant increases in both the 1.0 and 1.5 mg/kg supplementation groups (*p* < 0.001).

### 3.3. Effects of Arecoline on Electronic Tongue Taste Analysis and Fatty Acid Components in Muscle

**Figure 3:**
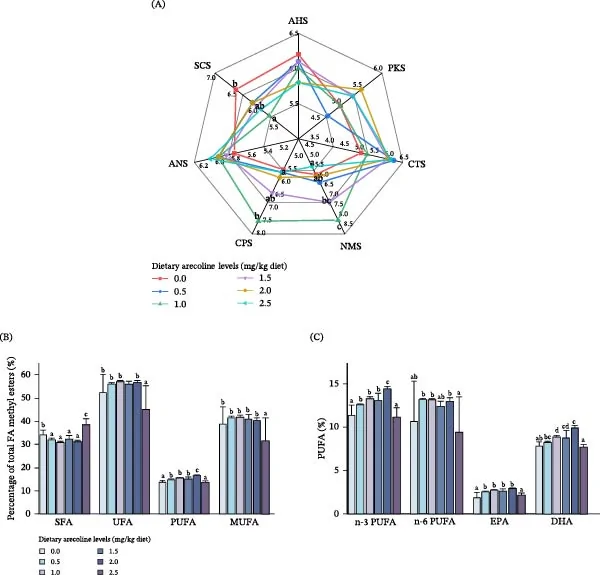
Effects of arecoline supplementation on the flavor characteristics and the profiles of fatty acids of grass carp muscle. (A) Radar chart of E‐tongue data; (B) the fatty acid (FA) profile; and (C) the polyunsaturated fatty acid (PUFA) profile. Values represent means ± SD (*n* = 6). Different letters indicate significant differences (*p* < 0.05). DHA, docosahexaenoic acid; EPA, eicosapentaenoic acid; MUFA, monounsaturated fatty acid; SFA, saturated fatty acid; UFA, unsaturated fatty acid.

The results of the electronic tongue test for muscle flavor analysis are shown in Figure 3A. The radar diagram shows the CTS, AHS, SCS, ANS, NMS, PKS, and CPS results of the muscle sample. Compared with control, the muscle of the 1.0 mg/kg group showed higher NMS (*p* = 0.013) and CPS (*p* = 0.046).

As illustrated in Figure 3B,C, arecoline supplementation (particularly between 0.5 and 2.0 mg/kg) significantly lowered the total saturated fatty acid (∑SFA) content (*p* < 0.001), while simultaneously elevating the total unsaturated fatty acids (∑UFA) (*p* = 0.004), including polyunsaturated fatty acids (∑PUFA) (*p* < 0.001), n‐3 PUFA (*p* < 0.001), EPA (*p* < 0.001), and DHA (*p* < 0.001) levels.

### 3.4. Effects of Arecoline on Parameters Related to Muscle Growth

**Figure 4:**
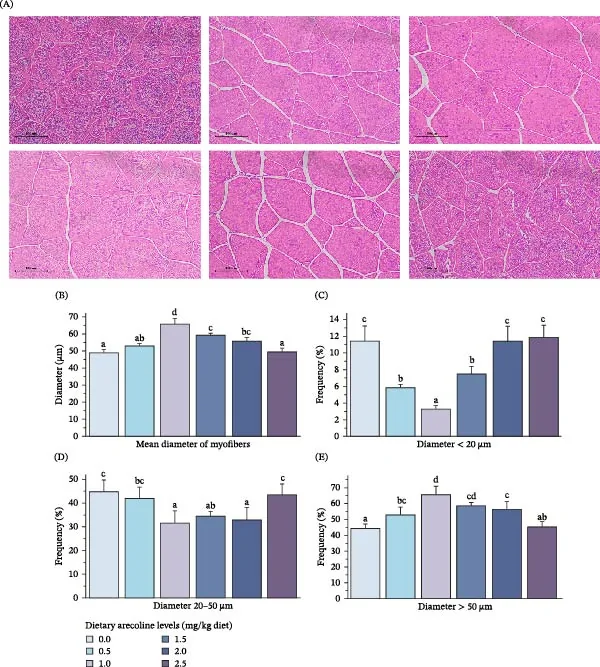
Effects of arecoline supplementation on the morphology and histology of the muscle of grass carp. (A) Hematoxylin‐eosin staining results in cross‐sections of fish muscle (200×); (B) mean diameter of muscle fiber (μm); (C) frequency (%) of distribution of myofibers in <20 μm diameter; (D) frequency (%) of distribution of myofibers in 20–50 μm diameter; and (E) frequency (%) of distribution of myofibers in >50 μm diameter. Values represent means ± SD (*n* = 3). Different letters indicate significant differences (*p* < 0.05).

As shown in Figure 4, the addition of arecoline increased myofiber diameter and the number of fibers over 50 μm, reaching a maximum at 1.0 mg/kg (*p* < 0.001). The number of muscle fibers with a diameter less than 20 μm significantly decreased when the arecoline level was increased to 0.5, 1.0, and 1.5 mg/kg (*p* < 0.001). Additionally, the occurrence of fibers with diameters between 20–50 μm was significantly lower in the 1.0, 1.5, and 2.0 mg/kg groups (*p* = 0.014).

**Figure 5:**
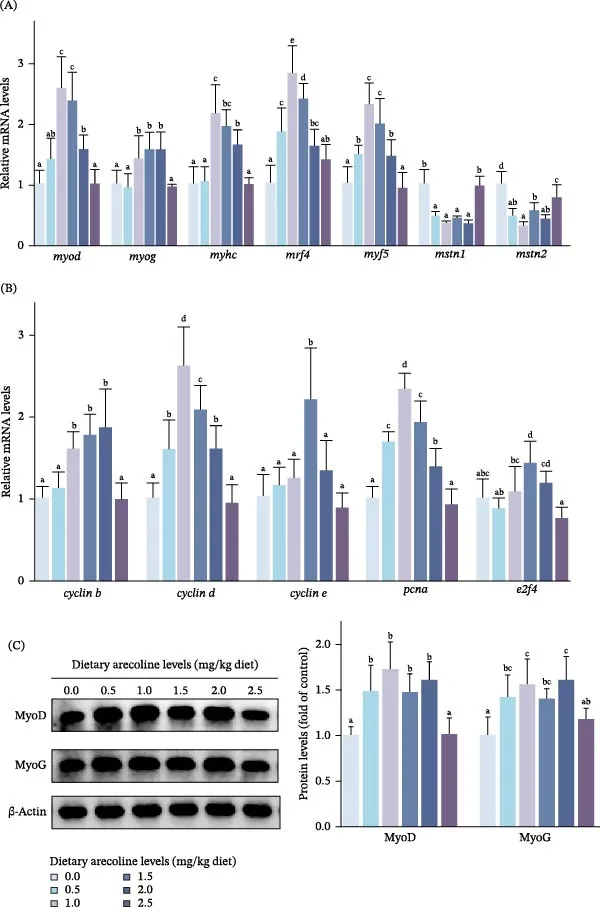
Effects of arecoline supplementation on parameters related to muscle growth in adult grass carp muscle. (A–B) Relative mRNA levels of factors associated with myoblast proliferation and differentiation pathways in muscle and (C) myoblast differentiation pathway‐related protein levels in muscle. Values represent means ± SD (*n* = 6). Different letters indicate significant differences (*p* < 0.05).

As illustrated in Figure 5A,B, compared with the control, significantly higher *myod*, *myhc*, *mrf4*, *myf5*, *cyclin d*, and *pcna* mRNA levels were found in 1.0 mg/kg group (*p* < 0.001). In addition, the *cyclin e* and *e2f4* levels were significantly higher in 1.5 mg/kg group (*p* < 0.001). The *myog* and *cyclin b* levels in the muscle were significantly increased in the 1.0, 1.5, and 2.0 mg/kg groups (*p* < 0.001) and plateaued across these three concentrations. The levels of *mstn1* and *mstn2* were significantly lower (*p* < 0.001) in muscles supplemented with 1.0 mg/kg arecoline compared to the 0.0 mg/kg. As shown in Figure 5C, both MyoD and MyoG protein levels exhibited significant increases when 0.5–2.0 mg/kg of arecoline was added to the diet (*p* < 0.001).

### 3.5. Effects of Arecoline on Parameters Related to Muscle Protein Synthesis

**Figure 6:**
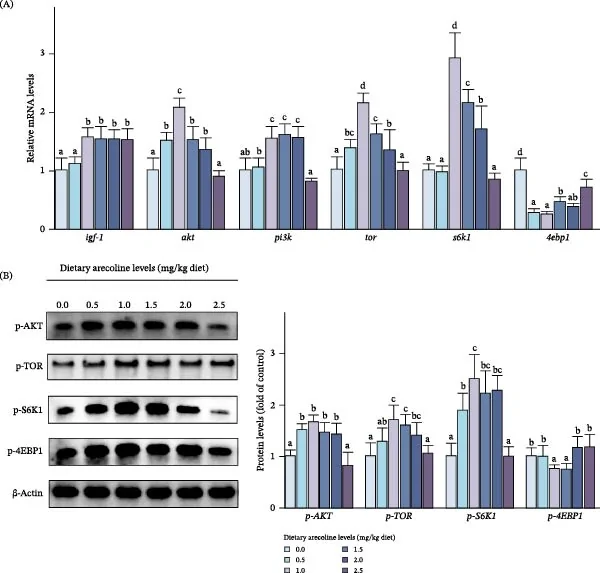
Effects of arecoline supplementation on parameters related to muscle protein synthesis in adult grass carp muscle. (A) Relative mRNA levels of factors associated with the protein synthesis pathway in muscle and (B) protein synthesis pathway‐related protein levels in muscle. Values represent means ± SD (*n* = 6). Different letters indicate significant differences (*p* < 0.05).

Figure 6A shows that the mRNA levels of *akt*, *tor*, and *s6k1* were significantly elevated in the 1.0 mg/kg group (*p* < 0.001). The level of *igf-1* mRNA gradually increased as the arecoline supplementation level rose to 1.0 mg/kg and then stabilized thereafter (*p* < 0.001). The *pi3k* mRNA level was significantly increased when arecoline was supplemented at 1.0–2.0 mg/kg in the diet (*p* < 0.001). In addition, the expression level of *4ebp1* was significantly reduced when the dietary arecoline concentration reached 0.5–2.5 mg/kg (*p* < 0.001). As presented in Figure 6B,C, compared with the 0.0 mg/kg group, diets supplemented with 1.0 mg/kg arecoline significantly increased p‐TOR and p‐S6K1 protein levels (*p* < 0.001). The p‐AKT protein level also showed significant increases when arecoline was supplemented at 0.5–2.0 mg/kg (*p* < 0.001). In contrast, the p‐4EBP1 protein level was significantly reduced in the 1.0 and 1.5 mg/kg supplementation groups (*p* < 0.001).

### 3.6. Effects of Arecoline on Parameters Related to Muscle Protein Degradation

**Figure 7:**
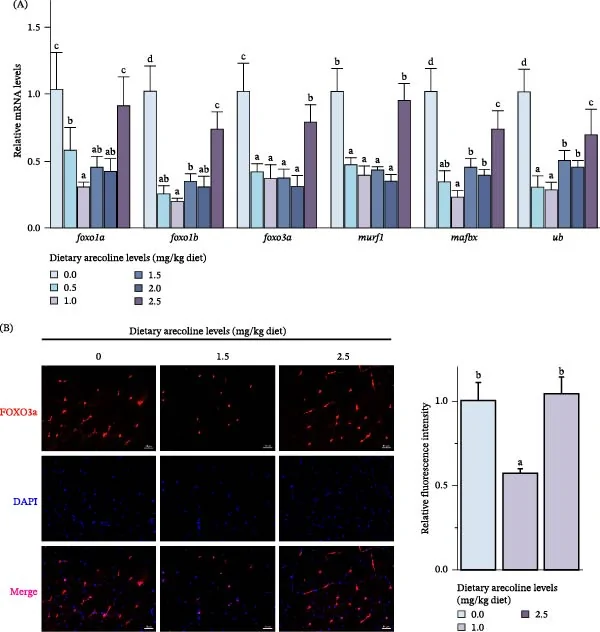
Effects of arecoline supplementation on parameters related to muscle protein degradation in adult grass carp muscle. (A) Relative mRNA levels of factors associated with ubiquitin protease pathway in muscle and (B) FOXO3a immunofluorescence staining and statistical results. Values represent means ± SD (*n* = 6). Different letters indicate significant differences (*p* < 0.05).

As presented in Figure 7A, compared with the control, the *foxo1a*, *foxo1b*, *foxo3a*, *murf1*, *mafbx*, and *ub* levels were significantly decreased in the 1.0 mg/kg group (*p* < 0.001). Immunofluorescence staining results for FOXO3a in Figure 7B were similar to its gene expression results (*p* < 0.001).

## 4. Discussion

As a bioactive alkaloid, arecoline has attracted attention for its potential to modulate growth and metabolism in aquatic animals. Our growth performance data (Table S5) confirmed that arecoline supplementation significantly improved feeding efficiency and growth metrics in grass carp [26], with no detectable residues in muscle tissue (Figure S1). Given that fish growth is closely linked to muscle development and that flesh quality is determined by physicochemical characteristics such as texture and nutritional value [2], we evaluated the effects of dietary arecoline on muscle quality and the associated signaling pathways.

### 4.1. Dietary Arecoline Improved Flesh Quality in Fish

The physicochemical properties and textural characteristics of meat are key determinants of quality, directly influencing consumer acceptability as well as the nutritional and commercial value of the product [27]. Cooking loss is an important indicator of WHC, and its reduction signifies improved flesh quality [28]. Mechanistically, during postmortem glycolysis, the breakdown of muscle glycogen into lactic acid directly drives the decline in muscle pH [29]. In the present study, arecoline supplementation reduced cooking loss and lactic acid content in grass carp muscle, suggesting that arecoline may attenuate the pH decline by inhibiting glycolytic flux or enhancing preslaughter glycogen storage, thereby improving WHC.

Muscle firmness is an important indicator of flesh quality in fish, commonly quantified by shear force [30], which reflects the structural integrity of muscle fibers and connective tissue. HYP, a characteristic component of collagen, directly influences collagen thermal stability and intermolecular cross‐linking, thereby determining muscle hardness and shear resistance. In this study, arecoline supplementation significantly increased the HYP content in grass carp muscle. Previous studies on Atlantic salmon have established that reduced muscle firmness is associated with decreased collagen content [10]; accordingly, our results suggest that arecoline may enhance muscle structural integrity by promoting collagen synthesis or inhibiting its degradation.

Crude protein, crude lipid, and moisture content are important indicators of the nutritional quality of fish muscle [10]. We have previously reported that arecoline supplementation improves the nutritional quality of grass carp muscle [26]. In the present study, we further observed a consistent increase in free amino acids in muscle, which may be attributable to the arecoline‐mediated regulation of amino acid transport or metabolism. Sanguorrhizine, a structurally similar alkaloid, has also been shown to induce comparable amino acid elevation in heat‐stressed broilers [31], suggesting that alkaloids may share conserved mechanisms for modulating amino acid metabolism. However, the precise molecular targets remain to be elucidated.

Flavor is an important dimension determining the market acceptability of aquatic products [32]. In this study, an electronic tongue system was employed to objectively evaluate taste attributes and minimize subjective bias in sensory assessment [33]. The results showed that arecoline supplementation significantly enhanced umami perception in muscle, consistent with the amino acid profile analysis: the arecoline‐treated groups (1.0–1.5 mg/kg) exhibited significantly higher levels of umami‐ and sweet‐tasting amino acids (Asp, Glu, Pro, Thr, Ser, and Gly) compared to the control, suggesting that arecoline may optimize the flavor profile through modulation of amino acid metabolism. Furthermore, fish are an excellent source of n‐3 PUFAs, particularly EPA and DHA [34]. In this study, arecoline supplementation increased muscle EPA and DHA contents, which may be attributed to the regulation of lipid metabolism‐related enzyme expression or activity or through enhanced uptake and incorporation of essential fatty acids. These improvements not only elevate the flavor quality of the fish but also enhance its value as a functional food product.

According to the study, appropriate arecoline supplementation could improve flesh quality, including physical and chemical quality, nutritional value, flavor quality, and health value‐related indexes.

### 4.2. Dietary Arecoline Promoted Muscle Growth in Fish

Fish growth is closely associated with muscle growth, which is driven by nutrient deposition within the muscle tissue and the dynamic processes of myofiber growth and remodeling [35], involving myoblast proliferation, differentiation, and continued myofiber hypertrophy [36]. Myofiber diameter distribution reflects fiber proliferation and hypertrophy [37]: a higher proportion of small‐diameter fibers (<20 μm) typically indicates active hyperplasia, while an increased proportion of fibers >50 μm in diameter is indicative of hypertrophy [38]. In this study, 0.5–2.0 mg/kg arecoline significantly altered the myofiber composition of grass carp, increasing the mean fiber diameter and the proportion of fibers >50 μm, while decreasing the proportions in the <20 μm and 20–50 μm ranges. Similar effects have been reported for other plant‐derived alkaloids, including capsaicin [39]. These changes suggest that arecoline primarily promotes myofiber hypertrophy rather than hyperplasia, thereby enhancing the muscle mass and overall growth in grass carp.

The growth and differentiation of fish myofibers are precisely regulated by a network of cyclin proteins and MRFs [40]. MRFs govern the division of skeletal myocytes, with MyoD playing a central role in myogenic cell proliferation [41], MyoG regulating myoblast fusion and differentiation, and MSTN inhibiting myocyte differentiation [42, 43]. MyHC serves as a marker of myofiber hyperplasia and hypertrophy [44]. In this study, dietary arecoline supplementation at 0.5–2.0 mg/kg upregulated the expression of *myod*, *myog*, *mrf4*, *myf5*, *myhc*, and *cyclins*, while downregulating *mstn* expression, consistent with the reported upregulation of MyoD by isoquinoline alkaloids from *Coptis japonica* in mouse C2C12 cells [45]. These molecular alterations suggest that arecoline may promote muscle development by modulating MRF expression to enhance myogenic differentiation and by stimulating cell cycle activity via cyclins. However, the causal relationships remain to be experimentally verified.

In summary, this study demonstrates that dietary arecoline supplementation is associated with muscle growth in fish, primarily manifested as myofiber hypertrophy, accompanied by upregulation of cyclins and MRFs and downregulation of MSTN expression, suggesting a potential mechanistic link that requires further validation.

### 4.3. Dietary Arecoline Improved the Muscle Protein Deposition

In addition to muscle growth, protein deposition is a major factor affecting fish flesh quality [46], which depends on the balance between protein synthesis and degradation [47, 48]. The TOR pathway is a central regulator of protein synthesis, promoting anabolic activity through phosphorylation of downstream effectors S6K1 and 4EBP1 [49, 50]. In this study, arecoline at 0.5–2.0 mg/kg upregulated *tor* and *s6k1* mRNA and phosphorylated protein levels while downregulating *4ebp1* levels, suggesting that arecoline may influence muscle protein synthesis via the TOR/S6K1/4EBP1 pathway. Furthermore, the PI3K/AKT pathway serves as another major anabolic axis [51], with TOR acting as its downstream effector, connecting both pathways into a unified regulatory network [52]. Our study showed that arecoline (0.5–2.0 mg/kg) increased *pi3k* and *akt* mRNA levels and p‐AKT protein levels and that arecoline at 1.0–2.5 mg/kg upregulated *igf-1* mRNA expression, which is known to activate the PI3K/AKT pathway [46]. Network pharmacology studies also support the potential modulatory role of arecoline on the PI3K/AKT pathway [53]. These findings suggest that arecoline may promote muscle protein deposition through the IGF‐1/PI3K/AKT/TOR/S6K1/4EBP1 pathway. However, this interpretation remains correlational and requires direct causal validation using pathway‐specific inhibitors or genetic approaches.

The UPP is a major catabolic mechanism responsible for targeted protein degradation [54]. Within this pathway, FOXO transcription factors (FOXO1 and FOXO3a) promote protein catabolism by upregulating muscle‐specific E3 ubiquitin ligases MURF1 and MAFBX [16]. Our study showed that arecoline at 0.5–2.5 mg/kg reduced the mRNA levels of *foxo1a*, *foxo1b*, *foxo3a*, *murf1*, *mafbx*, and *ub*, suggesting that arecoline may suppress UPP activity and reduce protein degradation. Consistently, the crude protein content was increased, although the difference was not statistically significant. (Table S5). Thus, arecoline may exert a dual effect—promoting synthesis (via TOR pathway) and inhibiting degradation (via UPP pathway)—to synergistically enhance protein deposition. Notably, AKT inhibits FOXO transcriptional activity by promoting its phosphorylation and subsequent nuclear exclusion [55]. The upregulation of *akt* and elevation of p‐AKT in arecoline‐treated groups in this study suggest that AKT activation may be involved in suppressing FOXO‐dependent protein degradation, although this mechanism requires further validation.

In summary, appropriate dietary arecoline supplementation enhances muscle protein synthesis. It reduces degradation in fish, a dual regulatory effect likely mediated through activation of the IGF‐1/PI3K/AKT/TOR/S6K1/4EBP1 anabolic pathway and suppression of FOXO‐mediated UPP activity. By coordinately regulating anabolism and catabolism, arecoline favors muscle growth and protein deposition. Notably, the observed alterations in gene and protein expression are correlational in nature, and definitive causal relationships await further validation through genetic or pharmacological approaches. Some mechanistic interpretations are extrapolated from mammalian studies and therefore require direct confirmation in piscine models. These limitations need to be addressed in future studies.

### 4.4. Optimal Supplementation of Arecoline in Adult Grass Carp Diet

**Figure 8:**
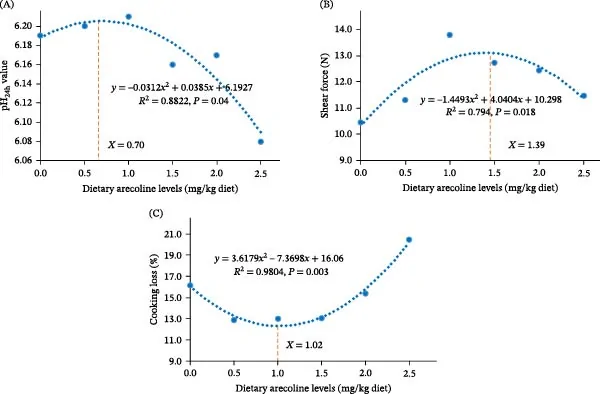
Quadratic regression analysis of pH_24h_ (A), shear force (N; B), and cooking loss (%; C) for the adult grass carp fed diets with graded levels of arecoline (mg/kg) for 9 weeks.

To establish the optimal dietary arecoline supplementation level for enhancing fish quality while ensuring safety, we systematically evaluated three critical quality parameters: (1) postmortem pH at 24 h (Figure 8A), reflecting acidification rate and its impact on flavor profile [56]; (2) shear force (Figure 8B), quantifying fish meat tenderness as a key determinant of consumer acceptability [46]; and (3) cooking loss (Figure 8C), indicating WHC that governs juiciness and mouthfeel [57]. Through this multiparametric analysis, we identified distinct optimal doses: 0.70 mg/kg for pH stabilization, 1.39 mg/kg for tenderness optimization, and 1.02 mg/kg for maximal water retention. Among the three parameters, the optimal dose based on pH (0.70 mg/kg) was lower than the other two, indicating that postmortem pH is more sensitive to arecoline supplementation. Previous studies have confirmed that low initial postmortem pH often leads to reduced WHC and softened texture, compromising the eating quality [58, 59]. Accordingly, it is reasonable to speculate that maintaining the supplementation level close to the pH‐based optimum may help delay postmortem pH decline and mitigate flesh quality deterioration. Multiresponse desirability optimization response surface analysis (Figure S2) further revealed that the comprehensive optimal addition level for the three quality parameters was 1.00 mg/kg at an overall desirability of 0.982, which is slightly higher than the optimal dose for pH alone, suggesting that a trade‐off among different quality dimensions should be considered in practical dose selection.

## 5. Conclusions

**Figure 9:**
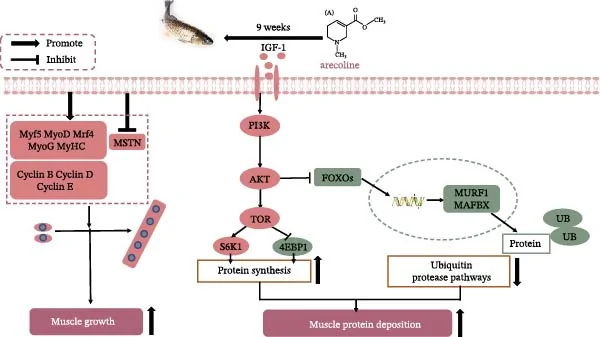
Dietary arecoline regulated the mechanism of muscle growth in grass carp.

In conclusion, as shown in Figure 9, dietary arecoline supplementation promotes muscle growth, improves flesh quality, and enhances protein deposition in grass carp. Appropriate arecoline levels improve the physicochemical properties (pH, hardness, and WHC), umami quality, and nutritional value (amino acids and fatty acids) of fish meat, thereby comprehensively enhancing the flesh quality. Meanwhile, arecoline increases muscle fiber diameter by modulating the TOR and FOXO signaling pathways, thereby promoting muscle protein deposition. Based on multiresponse desirability optimization via response surface methodology, with careful consideration of postmortem 24‐h pH, shear force, and cooking loss, the optimal dietary arecoline supplementation level for grass carp (608–1512 g) was determined to be 1.00 mg/kg. This finding provides a reference for precision nutritional regulation aimed at improving flesh quality in grass carp aquaculture production.

## Author Contributions

**Liangqin Wu:** methodology, formal analysis, investigation, data curation, writing – original draft. **Nao Yao:** methodology, investigation. **Weidan Jiang**: methodology, project administration. **Pei Wu:** methodology, project administration. **Yang Liu:** methodology, project administration. **Hongyun Zhang:** writing – review and editing, project administration. **Yaobin Ma**: methodology, project administration. **Changran Xu:** performed resources. **Qiyuan Chen:** performed resources. **Hequn Shi:** performed resources. **Xiaoqiu Zhou:** conceptualization, supervision, funding acquisition, resources. **Lin Feng:** writing – review and editing, conceptualization, supervision, methodology, project administration.

## Funding

This research was financially supported by the National Natural Science Foundation of China (Grant U23A20250), the National Key R&D Program of China (Grant 2023YFD2400600), the earmarked fund for CARS (Grant CARS‐45), and the Sichuan Innovation Team of National Modern Agricultural Industry Technology System (Grant SCCXTD‐2025‐15).

## Ethics Statement

The animal experimental procedures received official approval from the Institutional Animal Care and Use Committee of Sichuan Agricultural University (Approval ID: YN‐2020214026). This approval ensured that the research followed the highest ethical standards for animal welfare and care, in line with relevant regulations and guidelines governing the treatment and handling of animals in scientific research.

## Conflicts of Interest

The authors declare no conflicts of interest.

## Supporting Information

Additional supporting information can be found online in the Supporting Information section.

## Supporting information


**Supporting Information** Method details; The kits and instruments used for biochemical analysis (Table S1); Real‐time PCR primer sequences (Table S2); Target proteins, dilution factor, antibody cat. no. and antibody source of proteins selected for analyzing by western blotting (Table S3); Index formula for flesh rate and cooking loss of adult grass carp (Table S4); Effects of arecoline supplementation on the growth performance and muscle crude protein content of adult grass carp (Table S5); Effects of arecoline supplementation on fatty acid composition in the muscle of adult grass carp (% total fatty acids) (Table S6); Test report on the content of arecoline in the muscle of adult grass carp (Fig. S1); Response surface plot for multiresponse desirability optimization of arecoline addition level on pH, shear force, and cooking loss (Fig. S2).

## Data Availability

The authors confirm that the data supporting the findings of this study are available within the article, including its tables and figures. Data are available from the corresponding author upon reasonable request.
