# Supplementary material for: Dietary Arecoline Supplementation Enhances Flesh Quality, Muscle Growth, and Protein Deposition in Grass Carp (Ctenopharyngodon idella): Insights Into TOR and FOXO Signaling Pathways
Source: Aquac Nutr. 2026 Aug 26;2026:1084008. doi: 10.1155/anu/1084008 (PMC13507699; doi:10.1155/anu/1084008)
Supplement: Supplementary file 1 [file ANU-2026-1084008-s001.docx]

**Supplementary material**

**Method details**

**The pelletizer parameters**

pressure: within 100 MPa, speed: approximately 200 r/min.

**Electronic tongue activation and calibration methods**

First, passive activation was conducted, placing a special beaker containing distilled water in position 1 of the autosampling tray. The sensor array was then immersed manually in the distilled water for 30 minutes to stabilize. Subsequently, active activation and calibration procedures were performed using proprietary solutions from Alpha M.O.S., consisting of 0.01 mol/L NaCl, HCl, and monosodium glutamate (MSG), as outlined in the manufacturer's guidelines.

**Immunofluorescence staining**

For histological examination, tissue slices embedded in paraffin were first treated with an eco-friendly deparaffinizing agent, followed by inhibition of peroxidase activity using a 3% hydrogen peroxide solution. Tissue sections were subjected to overnight incubation with primary antibodies at 4°C, and a negative control was included to prevent nonspecific binding. Subsequently, secondary antibodies were applied for 1 hour under humid and dark conditions. DAPI was used for nuclear staining, and an antifluorescence quenching reagent was added to mount the sections. All stained samples were viewed using a Leica DMI4000B inverted fluorescence microscope under low-light conditions, applying identical imaging settings across samples. Fluorescence intensity was quantified only in cells showing positive staining.

**Table S1**

The kits and instruments used for biochemical analysis.

| Reagents/kits/Instruments | Manufacturer | Item No. |
| --- | --- | --- |
| benzocaine | Shanghai yuanye Bio-Technology Co., Ltd., Shanghai, China | S11036 |
| pH probe | Testo AG Company, Lenzkirch, Germany | Testo 205 pH meter |
| the Materials testing Instrument | Instron Corporation, Canton, MA, USA | INSTRON 4411 |
| Hydroxyproline kit | Nanjing Jiancheng Biotechnology Co., Ltd., Nanjing, China | A030-2-1 |
| Lactate kit |  | A019-2-1 |
| amino acid analyzer | Hitachi, Ltd., Tokyo, Japan | L-8900 and LA8080 |
| Gas chromatography analyzer | Shimadzu CO., LTD, Kyoto, Japan | GC-2010 Plus |

**Table S2** Real-time PCR primer sequences.

| Target gene | Primer sequence forward (5′ → 3′) | Primer sequence reverse (5′ → 3′) | Accession number | Amplification Efficiency, % | Amplification slopes |
| --- | --- | --- | --- | --- | --- |
| *igf-1* | GCTGCAGTTTGTGTGTGGAG | ATGCGATAGTTTCTGCCCCC | OL438919 | 97 | -3.39 |
| *pi3k* | AGTCAGTGCCTGTGGCTGAG | CGTGTCCATGACCTCAGAGC | KY763989 | 101 | -3.31 |
| *akt* | CCTGGTGATGAAGGAGCTGA | CTGTCAGAGAGCCTCCAGCA | KY763985 | 103 | -3.28 |
| *tor* | TCCCACTTTCCACCAACT | ACACCTCCACCTTCTCCA | JX854449 | 99 | -3.34 |
| *s6k1* | TGGAGGAGGTAATGGACG | ACATAAAGCAGCCTGACG | EF373673 | 94 | -3.48 |
| *4ebp1* | GCTGGCTGAGTTTGTGGTTG | CGAGTCGTGCTAAAAAGGGTC | KT757305 | 106 | -3.22 |
| *foxo1a* | GCATCTCATAGCCATGCCCT | CACCTCCAAGATGACCGGAG | KP325483 | 98 | -3.36 |
| *foxo1b* | CTCAACCTCATCTCGCCCAA | TCGGTATGGCGATTGGACTG | KP325484 | 105 | -3.24 |
| *foxo3a* | GCTGCGTAGTGATCCCATGATGTC | GGAGACTGTTGGAGATGCTGCTTC | (Zhou et al.2023) | 99 | -3.34 |
| *murf1* | TGTCTATGGACTACAGAGGAA | GGATTTCAAAGGAGGTTCAAG | (Zhou et al.2023) | 96 | --3.42 |
| *mafbx* | CGGACGAGATCTGGTTAGCC | GCTTGCGGATCTGTCTGTCT | (Zhou et al.2023) | 105 | -3.24 |
| *ub* | GCCAAGCGACACCATTGAG | GGATGTTGTAGTCGGACAG | (Zhou et al.2023) | 101 | -3.31 |
| *myog* | AGAGGAGGTTGAAGAAGGTC | GTTCCTGCTGGTTGAGAGA | JQ793897 | 95 | -3.45 |
| *myod* | CCCTTGCTTCAACACCAACG | TCTCCTCTCCCTCATGGTGG | GU218462 | 102 | -3.30 |
| *mrf4* | GAAAATCTGCTCCAACTACC | CGCTGCGTAAAATCTCCA | KT899334 | 98 | -3.36 |
| *myf5* | GTGCCTGTGCCTCATCTCCT | AATGCGTGGTTCACCTTCTTCA | GU290227 | 94 | -3.48 |
| *myhc* | GCTCATCACCACCAACCC | GCCTCCTCTGTGCCATCA | AB255030 | 104 | -3.26 |
| *mstn1* | GCAGGAGTCACGTCTTGGCA | GAGTCCCTCCGGATTCGCTT | KM874826 | 97 | -3.39 |
| *mstn2* | GAACAGGCTCCGAACATCAGC | GGTTGCAGTCCTTCTTCTCC | KM874827 | 102 | -3.30 |
| *β-actin* | GGCTGTGCTGTCCCTGTA | GGGCATAACCCTCGTAGAT | M25013 | 102 | -3.30 |
| *gapdh* | GTTACAAGGGAGAAGTTCACCAT | CCGGTAGACTCGACTACATACAG | (Dong et al.2010) | 101 | -3.31 |

**Table S3**

Target proteins, dilution factor, antibody cat. no. and antibody source of proteins selected for analyzing by western blotting.

| Target proteins | Dilution factor | Antibody cat. no. | Antibody source |
| --- | --- | --- | --- |
| MyoD | 1:1000 | A22059 | ABclonal, Wuhan, China;  Affinity BioReagents, Colorado, USA |
| MyoG | 1:1000 | A17427 |  |
| p-AKT Ser473 | 1:1000 | AF0016 |  |
| p-TOR Ser2448 | 1:1000 | AP0094 |  |
| p-4EBP1 T37/46 | 1:1000 | AP0030 |  |
| p-S6K1 T389 | 1:1000 | AP0564 |  |
| β-actin | 1:1000 | AF7018 |  |

**Table S4**

Index formula for flesh rate and cooking loss of adult grass carp (*Ctenopharyngodon idella*).

| Items | Formulas |
| --- | --- |
| Flesh rate | Flesh rate (%) = [carcass weight (g/fish) – fishbone weight (g/fish)]/FBW (g/fish) × 100 |
| Cooking loss | Cooking loss (%) = [muscle weight before cooking (g) –muscle weight after cooking (g)]/ muscle weight before cooking (g) × 100 |

**Table S5**

Effects of arecoline supplementation on the growth performance and muscle crude protein content of adult grass carp.

| Item | Dietary arecoline levels, mg/kg diet | | | | | | *P*-value |
| --- | --- | --- | --- | --- | --- | --- | --- |
|  | 0.0 | 0.5 | 1.0 | 1.5 | 2.0 | 2.5 |  |
| **Growth performance** |  |  |  |  |  |  |  |
| IBW, g/fish | 606.93 ± 1.85 | 609.07 ± 1.22 | 608.40 ± 2.62 | 608.53 ± 1.85 | 606.67 ± 2.44 | 607.60 ± 0.40 | 0.590 |
| FBW, g/fish | 1,285.87 ± 8.17^a^ | 1,389.33 ± 32.40^b^ | 1,512.53 ± 22.49^d^ | 1,437.33 ± 2.01^c^ | 1,390.4 ± 21.78^b^ | 1,255.2 ± 21.48^a^ | < 0.001 |
| PWG, % | 111.87 ± 1.99^a^ | 128.12 ± 5.71^b^ | 148.61 ± 3.61^d^ | 136.20 ± 0.44^c^ | 129.18 ± 3.08^b^ | 106.58 ± 3.54^a^ | < 0.001 |
| SGR, %/day | 1.19 ± 0.02^a^ | 1.31 ± 0.04^b^ | 1.45 ±0.02^d^ | 1.36 ± 0.01^c^ | 1.32 ± 0.02^b^ | 1.15 ±0.03^a^ | < 0.001 |
| FI, g/fish | 1,197.12 ± 1.76^b^ | 1,338.65 ± 1.04^d^ | 1,510.11 ± 1.05^f^ | 1,435.85 ± 1.17^e^ | 1,318.79 ± 1.36^c^ | 1,178.02 ± 1.65^a^ | < 0.001 |
| FE, % | 56.71 ± 0.76^ab^ | 58.29 ± 2.53^bc^ | 59.87 ± 1.45^c^ | 57.72 ± 0.09^abc^ | 59.43 ± 1.48^bc^ | 54.98 ± 1.89^a^ | 0.024 |
| **Muscle composition** |  |  |  |  |  |  |  |
| Crude protein (%) | 17.43 ± 2.22 | 17.48 ± 1.58 | 18.68 ± 1.29 | 18.29 ± 1.51 | 16.76 ± 1.74 | 16.08 ± 1.10 | 0.124 |

IBW = initial body weight; FBW = final body weight; PWG = percent weight gain; SGR = specific growth rate; FI = feed intake; FE = feed efficiency.

Values are means ± SD for three replicate groups, with 25 fish in each group, and mean values within the same row with different superscripts are significantly different (*P* < 0.05).

**Table S6**

Effects of arecoline supplementation on fatty acid composition in the muscle of adult grass carp (% total fatty acids).

| Item | Dietary arecoline levels, mg/kg diet | | | | | | SEM | *P*-values |
| --- | --- | --- | --- | --- | --- | --- | --- | --- |
|  | 0.0 | 0.5 | 1.0 | 1.5 | 2.0 | 2.5 |  | ANOVA |
| C14:1 | 0.07^abc^ | 0.07^bc^ | 0.08^c^ | 0.07^c^ | 0.06^a^ | 0.06^ab^ | 0.002 | 0.008 |
| C15:0 | 0.15^ab^ | 0.15^ab^ | 0.14^ab^ | 0.16^b^ | 0.13^a^ | 0.15^ab^ | 0.003 | 0.067 |
| C16:0 | 21.75^d^ | 20.98^bc^ | 20.39^ab^ | 21.20^cd^ | 20.20^a^ | 23.90^e^ | 0.224 | < 0.001 |
| C16:1 | 7.15^bc^ | 7.60^c^ | 7.95^c^ | 7.73^c^ | 6.43^ab^ | 6.24^a^ | 0.152 | < 0.001 |
| C18:0 | 6.78^b^ | 6.07^ab^ | 5.47^a^ | 6.56^ab^ | 6.51^ab^ | 8.28^c^ | 0.201 | < 0.001 |
| C18:1n9c | 31.00^b^ | 32.90^b^ | 33.10^b^ | 32.62^b^ | 33.12^b^ | 24.79^a^ | 0.852 | 0.019 |
| C20:0 | 0.11 | 0.10 | 0.11 | 0.12 | 0.11 | 0.13 | 0.004 | 0.299 |
| C18:3n6 | 0.14^ab^ | 0.16^b^ | 0.15^ab^ | 0.15^ab^ | 0.15^ab^ | 0.14^a^ | 0.002 | 0.144 |
| C20:1n9 | 0.11^a^ | 0.14^b^ | 0.14^b^ | 0.13^b^ | 0.12^b^ | 0.10^a^ | 0.003 | < 0.001 |
| C18:3n3 | 1.56^ab^ | 1.66^b^ | 1.66^b^ | 1.58^ab^ | 1.57^ab^ | 1.30^a^ | 0.041 | 0.098 |
| C22:0 | 1.03^b^ | 0.78^a^ | 0.85^a^ | 0.91^ab^ | 0.81^a^ | 1.34^c^ | 0.037 | < 0.001 |
| C20:3n6 | 1.15^b^ | 0.96^a^ | 0.96^a^ | 0.93^a^ | 1.05^ab^ | 1.37^c^ | 0.031 | < 0.001 |
| C20:3n3 | 0.14 | 0.13 | 0.13 | 0.13 | 0.13 | 0.13 | 0.002 | 0.855 |
| C20:5n3 (EPA) | 1.85^a^ | 2.50^b^ | 2.63^b^ | 2.63^b^ | 2.81^b^ | 2.10^a^ | 0.072 | < 0.001 |
| C22:6n3 (DHA) | 7.79^ab^ | 8.24^bc^ | 8.83^d^ | 8.73^cd^ | 9.91^e^ | 7.62^a^ | 0.147 | < 0.001 |
| ∑SFA | 34.11^b^ | 31.71^a^ | 30.64^a^ | 32.16^a^ | 30.95^a^ | 38.26^c^ | 0.504 | < 0.001 |
| ∑UFA | 52.20^b^ | 55.74^b^ | 57.02^b^ | 56.06^b^ | 56.59^b^ | 44.97^a^ | 1.093 | 0.004 |
| ∑PUFA | 13.38^a^ | 14.54^b^ | 15.23^b^ | 15.08^b^ | 16.40^c^ | 13.49^a^ | 0.198 | < 0.001 |
| ∑MUFA | 38.82^b^ | 41.21^b^ | 41.78^b^ | 40.98^b^ | 40.19^b^ | 31.48^a^ | 0.989 | 0.015 |
| ∑n3 | 11.34^a^ | 12.53^b^ | 13.25^b^ | 13.07^b^ | 14.43^c^ | 11.15^a^ | 0.222 | < 0.001 |

ΣSFA = total saturated fatty acids; ΣUFA = total unsaturated fatty acids; ΣMUFA = total monounsaturated fatty acids; ΣPUFA = total polyunsaturated fatty acids; EPA = eicosapentaenoic acid; DHA = docosahexaenoic acid.

**Fig. S1** Test report on the content of arecoline in the muscle of adult grass carp.

**Fig. S2** Response surface plot for multi-response desirability optimization of arecoline addition level on pH, shear force, and cooking loss, with overall desirability of 0.982 and optimal addition level of 1.00 mg/kg.
